# The Role of Neuromodulators in Cortical Plasticity. A Computational Perspective

**DOI:** 10.3389/fnsyn.2016.00038

**Published:** 2017-01-10

**Authors:** Victor Pedrosa, Claudia Clopath

**Affiliations:** ^1^Department of Bioengineering, Imperial College LondonLondon, UK; ^2^CAPES Foundation, Ministry of Education of BrazilBrasilia, Brazil

**Keywords:** neuromodulation, noradrenaline, acetylcholine, dopamine, synaptic plasticity, computational modeling

## Abstract

Neuromodulators play a ubiquitous role across the brain in regulating plasticity. With recent advances in experimental techniques, it is possible to study the effects of diverse neuromodulatory states in specific brain regions. Neuromodulators are thought to impact plasticity predominantly through two mechanisms: the gating of plasticity and the upregulation of neuronal activity. However, the consequences of these mechanisms are poorly understood and there is a need for both experimental and theoretical exploration. Here we illustrate how neuromodulatory state affects cortical plasticity through these two mechanisms. First, we explore the ability of neuromodulators to gate plasticity by reshaping the learning window for spike-timing-dependent plasticity. Using a simple computational model, we implement four different learning rules and demonstrate their effects on receptive field plasticity. We then compare the neuromodulatory effects of upregulating learning rate versus the effects of upregulating neuronal activity. We find that these seemingly similar mechanisms do not yield the same outcome: upregulating neuronal activity can lead to either a broadening or a sharpening of receptive field tuning, whereas upregulating learning rate only intensifies the sharpening of receptive field tuning. This simple model demonstrates the need for further exploration of the rich landscape of neuromodulator-mediated plasticity. Future experiments, coupled with biologically detailed computational models, will elucidate the diversity of mechanisms by which neuromodulatory state regulates cortical plasticity.

## 1. Introduction

Cortical circuits are modified by experience. It is widely thought that such modifications enhance the representations of behaviorally important sensory stimuli, such as natural scenes for the visual cortex, or speech for the auditory cortex. These modifications can lead to the development or refinement of receptive fields in cortical neurons through synaptic plasticity. Across many species, the amount of cortical plasticity has been shown to depend on age. In juvenile mice, for example, tuning curves can shift from responding maximally to a preferred stimuli to responding maximally to a training stimulus (Dorrn et al., 2010). This is not thought to require neuromodulation and, interestingly, is associated with a state of unbalanced excitation and inhibition. Conversely, experimental data from adults suggest that sensory stimulation is not sufficient to induce a change in receptive fields. Experiments in adult mice suggest that activation of neuromodulatory systems is also necessary for this change (Bear and Singer, 1985; Bakin and Weinberger, 1996; Kilgard and Merzenich, 1998; Shulz et al., 2000; Gu, 2002; Ma and Suga, 2005; Froemke et al., 2007; Drever, 2011; Chun et al., 2013; Martins and Froemke, 2015). In primary auditory cortex, Froemke et al. (2007) observed that repeated exposure to a training sound frequency is not sufficient to evoke experience-dependent plasticity in adult rats. However, the tuning curve shifts to the training frequency if the stimulus is paired with cholinergic stimulation (from Nucleus Basalis). Interestingly, the stability of receptive fields is thought to be associated with the balance between excitation and inhibition, which is observed across many regions in the adult brain (Destexhe and Sejnowski, 2003; Shu et al., 2003; Wehr and Zador, 2003; Haider et al., 2006; Froemke et al., 2007; Okun and Lampl, 2008; Froemke et al., 2013; Graupner and Reyes, 2013; Xue et al., 2014). Experiments indicate that some neuromodulators act to disrupt this balance (Froemke et al., 2007, 2013; Letzkus et al., 2011), enabling cortical plasticity. These neuromodulatory systems may be responsible for communicating the behavioral context of sensory stimuli to other brain regions (Shulz et al., 2000; Gu, 2002).

Neuromodulators are observed to induce different effects in neural circuits. One main neuromodulatory effect is to gate plasticity by modifying the spike-timing-dependent plasticity (STDP) learning window (Bissière et al., 2003; Couey et al., 2007; Seol et al., 2007; Caporale and Dan, 2008; Lin et al., 2008; Pawlak and Kerr, 2008; Shen et al., 2008; Zhang et al., 2009; Pawlak et al., 2010). For example, in lateral amygdala, activation of D2 dopamine receptors was shown to be necessary to induce long-term potentiation (LTP) (Bissière et al., 2003). While in dorsal striatum, dopamine signaling via D1/D5 receptors is required for both long-term potentiation and long-term depression (LTD) (Pawlak and Kerr, 2008). In prefrontal cortex layer 5 pyramidal neurons, nicotine was shown to be able to reverse LTP into LTD (Couey et al., 2007). In visual cortex, the combined action of acetylcholine and noradrenaline is necessary for standard STDP, whereas the action of noradrenaline alone was shown to reverse LTD into LTP, and acetylcholine alone allows only LTD (Seol et al., 2007). Therefore, depending on the brain region and, possibly, the stimulation protocol, neuromodulatory signaling can completely reshape STDP learning windows. Additionally, dopamine has been shown to be important for reinforcement learning (Schultz, 2002). Unlike previous work on dopamine and reinforcement learning, we focus this perspective article on other less explored neuromodulatory effects in an unsupervised learning scheme.

Neuromodulators have also been shown to upregulate neuronal activity. For example, cholinergic stimulation is known to lower feedforward inhibition (Woody and Gruen, 1987; Metherate et al., 1992; Xiang, 1998; Froemke et al., 2007, 2013). Similarly, noradrenaline is known to trigger a disinhibitory effect (Kuo and Trussell, 2011). It has been shown that stimulation of Locus Coeruleus, the main source of noradrenaline, reduces tonic inhibition in auditory cortex (Martins and Froemke, 2015). These disinhibitory mechanisms are thought to be essential for adult cortical plasticity (Hensch, 2005; Letzkus et al., 2011; Kuhlman et al., 2013). Indeed, a computational study by Clopath et al. (2016) demonstrated that a disinhibitory gate is required for adult cortical plasticity. These findings indicate that a disinhibited system promotes learning, consistent with recent experimental work (Letzkus et al., 2011; Kuhlman et al., 2013).

In this perspective article, we review the possible effects of neuromodulators by using a simple computational model of a plastic feedforward network. First, we hypothesize four different learning windows that would result from the action of different neuromodulators. Then we show the consequences of these rules on receptive field plasticity. We verify that an antisymmetric STDP rule allows for receptive field development, whereas a rule with more potentiation allows for a greater modification of sensory representation. Finally, we compare the effect of upregulating the learning rate to the effect of upregulating activity and show that they are not necessarily equivalent. In the simple model, upregulating activity can lead to either a sharpening or a broadening of receptive field tuning. Upregulating the learning rate, however, only amplifies the existing structure.

## 2. Results

**Figure 1:**
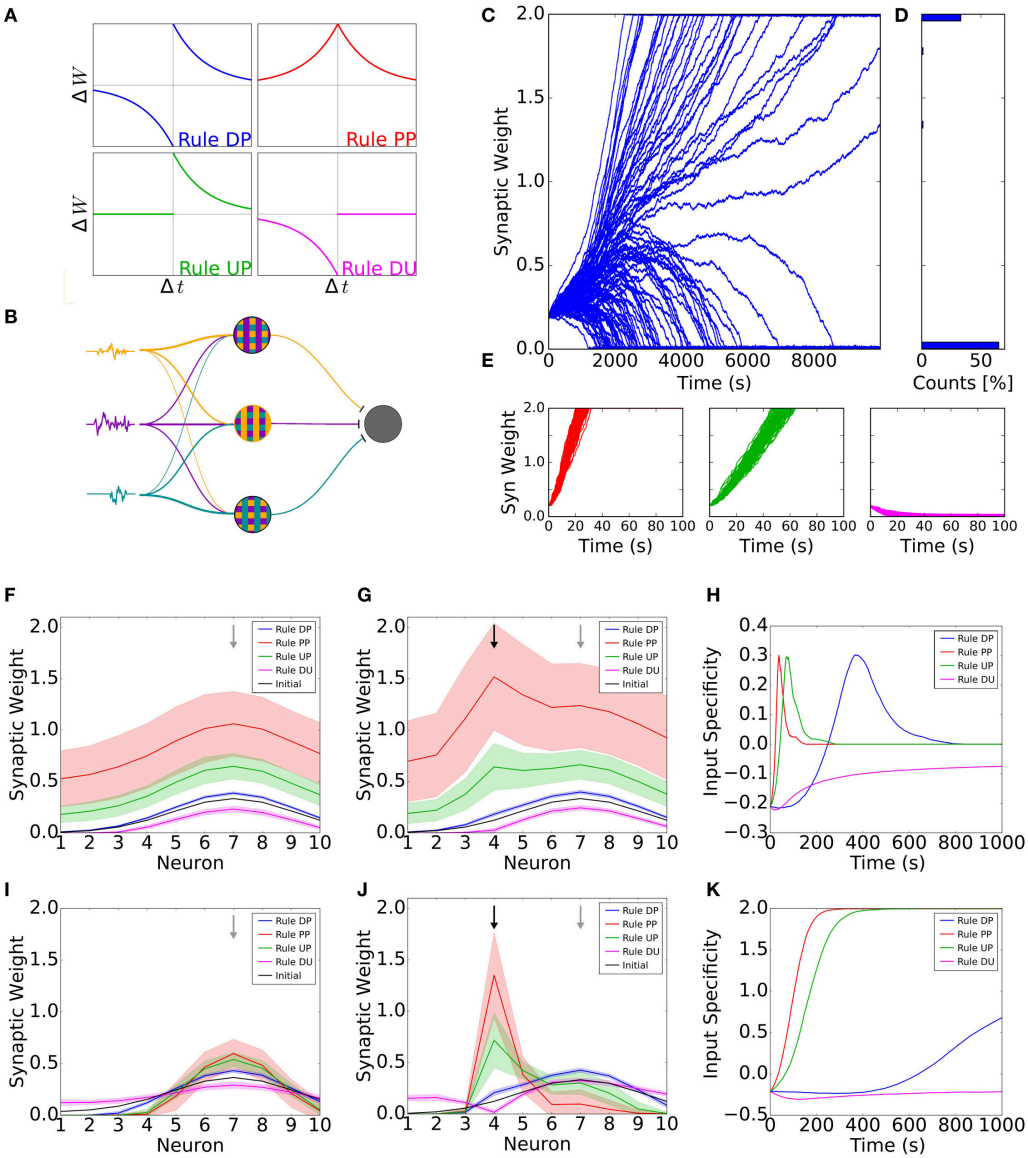
**Receptive field plasticity under the effect of neuromodulation. (A)** Diagram showing the four learning windows. Each learning window shows the change in synaptic strength (Δ*W*) as a function of the difference between the post- and presynaptic spike times (Δ*t* = *t_post_*−*t_pre_*). Blue, rule DP (Depression-Potentiation); red, rule PP (Potentiation-Potentiation); green: rule UP (Unchanged-Potentiation); pink, rule DU (Depression-Unchanged). **(B)** Network diagram. Firing probabilities (signals, colored traces) are independently generated and each neuron's firing probability is determined by a weighted sum of these signals. Each signal can be understood as one specific sensory feature, such as one particular tone for auditory stimulation or one particular orientation for visual stimulation. The input neurons project to one common postsynaptic neuron (gray circle). **(C–E)** Evolution of synaptic weights for the different learning rules. **(C)** Evolution of weights for a simulation with 100 presynaptic neurons projecting to one postsynaptic neuron. The excitatory weights follow the DP rule with the amplitude for depression slightly greater than the amplitude for potentiation. The small difference in amplitude is enough to generate bimodal distribution of weights. **(D)** Final distribution of weights in **(C)**. The synaptic weights are in the vertical axis and the counts of synaptic weights in each interval are in the horizontal axis. **(E)** First 10 seconds of the evolution of weights for plasticity rules PP, UP and DU (red, green and pink, respectively). The weights quickly achieve the upper or lower bounds. **(F–K)** Simulation of a network with 10 presynaptic neurons. The excitatory connections follow the four STDP rules in **(A)**. **(F)** Final synaptic weights for each input neuron, except black line, which is the initial weights. The initial receptive field was tuned to input neuron 7 (grey arrow). All the inputs had the same intensity. **(G)** Final synaptic weights for each input neuron when stimulus 4 (training input, black arrow) is 100% stronger than the other stimuli. Black curve shows the initial weights, tuned to input neuron 7 (grey arrow). **(H)** Difference between the synaptic weight from input neuron 4 (training input) and the weight from input neuron 7 (initial preferred input) as a function of time. We call this difference ‘input specificity’. **(I–K)** Same as **(F–H)** but for a system in which the excitatory weights are also constrained by a normalization rule. In Figures **(F–K)**, curves show the mean averaged over 100 trials and shaded areas represent one standard deviation from the mean.

To illustrate the effect of neuromodulation in cortical plasticity, we use four possible STDP learning rules for excitatory synapses (Figure 1A). Each one of the STDP rules can be thought of as the action of a specific neuromodulatory state. The first rule is the standard antisymmetric STDP rule, in which a presynaptic spike preceding a postsynaptic action potential leads to potentiation of synaptic connections, whereas the reverse leads to depression. We refer to this rule as the Depression-Potentiation (DP) rule (Figure 1A, blue curve). Although widely observed in neocortical neurons of juvenile animals (Markram et al., 1997; Feldman, 2000; Sjöström et al., 2001; Nevian and Sakmann, 2006), the DP rule seems to be neuromodulator dependent in adults. This rule has been observed in visual cortex when both noradrenaline and acetylcholine are present (Seol et al., 2007), whereas in dorsal striatum it can be observed under activation of D1/D5 (dopamine-specific) receptors (Pawlak and Kerr, 2008). The second rule is a symmetrical STDP rule, in which all pairs of pre- and postsynaptic spikes lead to potentiation, regardless of their order. As such, we refer to this as the Potentiation-Potentiation (PP) rule (Figure 1A, red curve). This rule can be observed in adult visual cortex under the activation of β-adrenergic (noradrenaline-specific) receptors (Seol et al., 2007), and in hippocampal neurons under the effect of dopamine (Zhang et al., 2009). For the third rule, only presynaptic spikes followed by postsynaptic action potentials elicit synaptic weight changes, leading to potentiation. Thus, we refer to this as the Unchanged-Potentiation (UP) rule (Figure 1A, green curve). This rule can be associated with dopaminergic action via D2 receptors in Lateral amygdala (Bissière et al., 2003). Lastly, the fourth rule states that synaptic weights are weakened every time a postsynaptic spike precedes presynaptic action potentials, and is unchanged otherwise and hence we refer to this as the Depression-Unchanged (DU) rule (Figure 1A, pink curve). This rule has been reported in prefrontal cortex under nicotinergic modulation (Couey et al., 2007).

We simulate a feedforward network, in which a set of presynaptic input neurons project onto one postsynaptic neuron (Figure 1B). Input neurons fire with a time-varying firing rate with Poisson statistics. Neighboring input neurons have correlated activity, as for example neurons with similar frequency/orientation (sensory feature) preference in auditory/visual cortex (for more details, see Section 4 and Supplementary Figure 1). Using this network, we illustrate the receptive field formation and adaptation under different learning rules, which are shaped by different neuromodulatory states.

### 2.1. Standard STDP leads to symmetry breaking

Hebbian learning rules, when associated with a competitive mechanism, are known to induce symmetry breaking of synaptic weights, i.e., some weights become strong and some weights become weak. However, it is well known that even without explicit competition, it is possible to induce symmetry breaking. This happens when the STDP rule is such that the depression component of the STDP learning window is larger than the potentiation component (i.e., the integral of the learning window is negative) (Song et al., 2000). To illustrate the behavior of synaptic weights for the four different learning rules, we first simulate a network composed of 100 presynaptic neurons with all synaptic weights initially set at the same value. The DP rule can be modified to ensure that the amount of depression is slightly higher than the amount of potentiation (by increasing the amplitude of depression by 2%, as observed in several experiments (Bi and Poo, 1998; Feldman, 2000). We observe that, after some time, some of the weights get completely depressed whereas the remaining get completely potentiated, i.e., the weights go to their upper and lower bounds (Figures 1C,D). This happens because inputs with strong connections can more easily induce a postsynaptic action potential. As a consequence, the spiking activities of strongly connected inputs are more correlated with the activity of the postsynaptic neuron, resulting in a further strengthening of weights. For those inputs with weak connections, there is almost no correlation between their activity and that of the postsynaptic neuron. These low correlations ensure the learning window is equally sampled so that the asymmetry of potentiation and depression results in depression of these weak synaptic weights. This effect was formally derived in several studies (e.g., Kempter et al., 1999; Song et al., 2000; Gilson et al., 2009; Babadi and Abbott, 2016). For the remaining learning rules, synaptic weights are either all potentiated or all depressed (Figure 1E).

Taken together, the DP rule is the only one which allows for the emergence of receptive fields in this simple model. This rule can be associated with the combined action of noradrenaline and acetylcholine in visual cortex (Seol et al., 2007). This model suggests that development of receptive fields in this region can be facilitated through the action of these neuromodulators. The first section of this perspective considered the neuromodulatory state for receptive field formation. Next, we will show that the neuromodulatory state for receptive field adaptation might be different.

### 2.2. The stronger the potentiation, the faster the receptive field plasticity

In order to demonstrate the effect of different neuromodulatory states on the stability of receptive fields, we first consider the case where all inputs have on average the same firing rate. We assume a network with 10 inputs in which the postsynaptic neuron is already tuned to a preferred stimulus, i.e., it has a stronger weight for input 7 than for other inputs. For the DP rule (standard STDP) we observe a small increase in all synaptic weights, even though the amounts of potentiation and depression are the same. Synaptic changes are larger for synapses that were initially stronger in these simulations (Figure 1F, blue curve). The activity of presynaptic neurons increases the probability of postsynaptic action potentials. Therefore, events in which presynaptic neurons fire before the postsynaptic neuron (while close in time) are more likely to occur than the opposite, as shown previously (Kempter et al., 1999; Song et al., 2000; Gilson et al., 2009). The PP rule, with the largest amount of potentiation, results in the largest change of receptive field (Figure 1F, red curve), whereas the UP rule generates an intermediate increase (Figure 1F, green curve). For the DU rule, we observe a small decrease of all synaptic weights (Figure 1F, pink curve). Therefore, we observe that the final receptive field remains tuned to the initial tuning frequency for all learning rules (Figure 1F).

It has been demonstrated that neuromodulation can facilitate plasticity in different systems (Bear and Singer, 1985; Bakin and Weinberger, 1996; Shulz et al., 2000; Gu, 2002; Froemke et al., 2007; Chun et al., 2013; Martins and Froemke, 2015). In this section, we want to illustrate how different learning rules, mediated by different neuromodulatory states, affect receptive field plasticity. To do this, we over-represent one input (input 4), called the training input, by increasing the firing rate of one input neuron and its neighbors. This corresponds to stimulating one sensory feature excessively—e.g., by the repeated presentation of one tone (for auditory stimulus) or one orientation (for visual stimulus). We observe a shift in the receptive field towards the training input for learning the PP and UP rules (Figure 1G, red and green curves), which are potentiation-only rules. For the DP rule, we observe a small increase in the connection from the training input. After 40 s, the peak of the receptive field is still at the initially preferred input. As such, the receptive field did not shift to the training input (Figure 1G, blue curve). When applying the DU rule, the stronger activation of the training input leads to a stronger depression of the corresponding synapse (Figure 1G, pink curve). When we compare the strength of the synapse from the training input with the one from input 7 (the initially preferred input), we observe that the rules with only potentiation lead to fast receptive field plasticity towards the new preferred stimulus. Since the weights are bounded, this shift in the preferred input is transient and the postsynaptic neuron is untuned at the end of the simulation (Figure 1H). The antisymmetric DP rule leads to a slower shift yet achieving, on average, the same value of input specificity as the PP and UP rules (Figure 1H). The DU rule forces all the weights to decrease until they reach the lower bound value. Therefore, the difference between the neuron's response to the training input and the initially preferred input slowly converges to zero (Figure 1H, pink curve). Not surprisingly, only rules with potentiation lead to a receptive field shift towards the training input. Additionally, rules with a larger amount of potentiation result in a faster receptive field shift.

In the model so far, there was no explicit competition mechanism between weights for any of the learning rules implemented until now. We now want to illustrate whether such a mechanism would facilitate or obstruct receptive field plasticity. To address this question, we use a normalization rule together with the STDP learning rules. Again, we first consider the case where all the inputs have on average the same firing rate. For rules DP, PP and UP, we observe a narrowing of the receptive field tuning, whereas rule DU leads to a flattening (Figure 1I). When one stimulus is stronger, the receptive field is shifted towards the strongest stimulus for rules DP, PP and UP, whereas rule DU results in a weakening of the connection associated with this stimulus until it reaches the lower bound (Figure 1J). This receptive field shift is slower than for the case without normalization but can lead to a strong receptive field tuned to the new stimulus (Figure 1K).

In summary, the learning rule with the largest amount of potentiation is more efficient for receptive field plasticity, both with and without normalization. This learning rule, rule PP, can be associated with the action of noradrenaline on visual cortex (Seol et al., 2007). Therefore, our results suggest that noradrenaline is a good candidate for facilitating receptive field plasticity in this brain region.

### 2.3. Modulation of neuronal activity and learning rates have different effects in receptive field plasticity

Neuromodulation has been shown to affect many processes. Primarily, it has been shown to upregulate activity (e.g., by disinhibition—Froemke et al., 2007; Martins and Froemke, 2015) or to gate plasticity (e.g., by changing the learning rule—Bissière et al., 2003; Couey et al., 2007; Seol et al., 2007; Pawlak and Kerr, 2008). Intuitively, these two effects seem to be equivalent, since synaptic weight changes from Hebbian learning can be modeled as a product of the learning rate and neuronal activity. But is this really the case? Here we demonstrate in our simple computational model that upregulating either the learning rate or neuronal activity leads to different synaptic weight changes. To this end, we model a feedforward network with only one presynaptic and one postsynaptic neuron. The synaptic weights are updated according to learning rule DP (Figure 1A) with a learning rate amplitude α, and the presynaptic neuron fires with firing rate ν. We add an extra noise current to the postsynaptic neuron in order to ensure postsynaptic firing at 10 Hz when the input neuron is kept silent.

**Figure 2:**
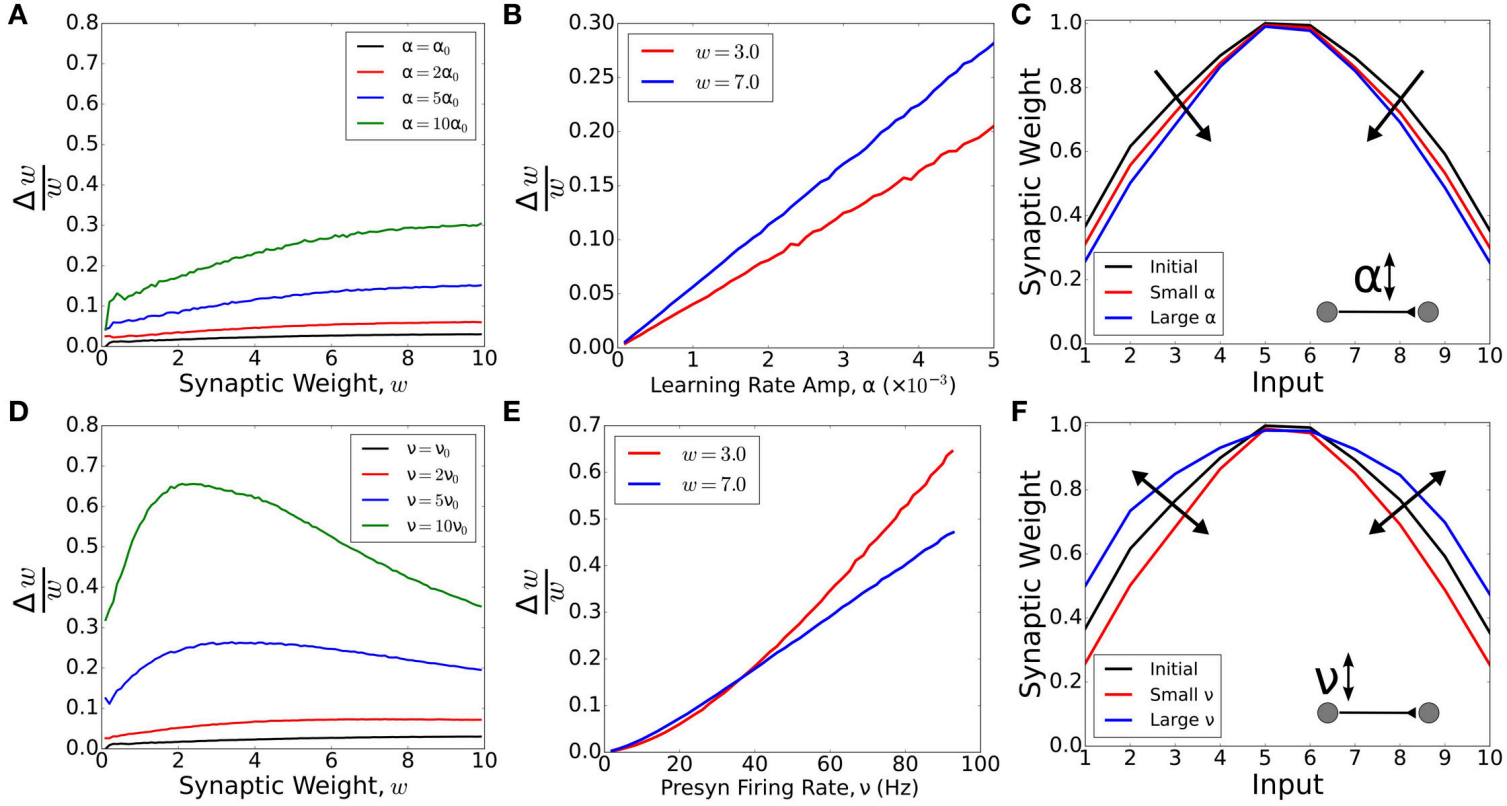
**Modulation of activity vs modulation of learning rate**. A network with one pre- and one postsynaptic neuron was simulated (in A, B, D and E). The synaptic weight changed following a standard STDP rule with amplitude α and the presynaptic neuron fired with firing rate ν. For synaptic weight *w* = 0, the postsynaptic neuron fired with firing rate ~10 Hz. **(A)** Ratio between the synaptic change and the synaptic weight as a function of the weight for different values of α, with α_0_ = 0.0005 and presynaptic firing rate ν = 10 Hz. **(B)** Ratio between the synaptic change and the synaptic weight as a function of the amplitude of learning for *w* = 3.0 (red) and *w* = 7.0. In both cases, the presynaptic firing rate was set to ν = 10 Hz. **(C)** Synaptic weights for a feedforward network with 10 presynaptic neurons and one postsynaptic neuron. The final synaptic weights were calculated for low presynaptic neuronal activity (ν = 1 Hz) and two values of learning rate: α = 0.01 (small α, red curve) and α = 0.02 (large α, blue curve). The initial and final tuning curves were re-scaled by dividing all the tuning curves by their respective maximum weights. The increase in α always sharpens the receptive field tuning (for low presynaptic activity). **(D)** Ratio between the synaptic change and the synaptic weight as a function of the weight for different values of ν, with ν_0_ = 10 Hz and the amplitude of learning α = 0.0005. **(E)** Ratio between the synaptic change and the synaptic weight as a function of the presynaptic neuronal firing rate for *w* = 3.0 (red) and *w* = 7.0. In both cases, the amplitude of learning was set to α = 0.0005. **(F)** Synaptic weights for a feedforward network with 10 presynaptic neurons and one postsynaptic neuron. The final synaptic weights were calculated for learning rate α = 0.02 and two values of presynaptic activity: ν = 1 Hz (small ν, red curve) and ν = 10 Hz (large ν, blue curve). The initial and final receptive fields were re-scaled by dividing all the tuning curves by their respective maximum weights. The modulation of neuronal activity, ν, can lead to either a sharpening or a flattening of receptive field tuning, depending on the value of ν. In Figures **(A,B,D,E)**, the curves are averages over 200 trials. In Figures **(C,F)**, the curves are averages over 50 trials.

First, we ask how plasticity depends on the synaptic weight, and whether the modulation of learning amplitude can alter this dependence (Figures 2A–C). We calculate the ratio between the synaptic weight change, Δ*w*, and the synaptic weight, *w*, as a function of the weight. If the change is proportional to the weight, the ratio is constant. However, for small values of presynaptic activity, ν, strong weights increase relatively faster compared with weak weights, regardless of the value of the learning amplitude, α (Figure 2A). We observe that Δ*w*/*w* is proportional to the learning amplitude (Δ*w*/*w* = *kα*) and the proportionality constant, *k*, is higher for strong weights (Figure 2B). As such, strong weights change relatively faster than weak weights and the modulation of learning can only amplify or reduce this difference, but not reverse it.

Having shown how plasticity depends on the learning rate, we now ask whether there is a similar dependence on neural activity (Figures 2D–F). We observe that, for large values of ν, the relative weight increase (Δ*w*/*w*) does not increase with weight. Instead, small weights can grow faster than large weights for large enough values of ν (Figure 2C). For large weights, the modulation of neuronal activity has a similar effect to the modulation of learning rate. However, for weak weights, the modulation of neuronal activity can have a stronger effect (Figure 2D). Therefore, by controlling the activity of the presynaptic neuron, it is possible to shift from a scenario where strong weights learn faster to a scenario in which weak weights learn faster.

In summary, regulation of activity can lead to a scenario where weak weights learn relatively faster than strong weights. In other words, the regulation of activity can control whether the receptive field of a neuron is either sharpened of broadened. To demonstrate this, we simulate a feedforward network with 10 presynaptic neurons for two levels of presynaptic activity. For low activity, we observe a sharpening, whereas high activity leads to a broadening of the receptive field (Figure 2F, Supplementary Figure 2). The upregulation of learning rate, on the other hand, can only amplify receptive field changes in our model. Therefore, for low firing rates, the regulation of learning rate will always lead to a sharpening of the receptive field tuning, regardless of the learning rate amplitude (Figure 2C, Supplementary Figure 2). These two modulation mechanisms act independently and do not disturb each other in our model (Supplementary Figure 2). The same behavior is observed when we use a more realistic, non-linear STDP model such as the triplet model (Pfister and Gerstner, 2006) (Supplementary Figure 3). Due to this large qualitative difference, more experiments are needed in order to identify how much, in which proportion, and when neuromodulation affects either learning, neural activity, or a combination of both.

## 3. Discussion

In this perspective article, we used four different learning rules, each associated with one or more neuromodulatory states, to illustrate how neuromodulation can affect receptive field plasticity. In order to explore the effects of different neuromodulatory states, we implemented these four learning rules in a feedforward network. As expected (Song et al., 2000), we observed that receptive field development was only possible for one of these rules. This learning rule (DP) can be associated with the combined action of noradrenaline and acetylcholine in visual cortex, or the action of dopamine via D1/D5 receptors in dorsal striatum. It suggests that these neuromodulators can be important to the development of receptive fields in these brain regions, under the assumption that STDP is the dominant player in cortical plasticity. In our analysis, we also asked what would be the best rule to change a receptive field once it is formed. To this end, we combined each learning rule with trained input. This simulates the association of a neuromodulatory state paired with a stimulus (e.g., a tone frequency, or an oriented bar). We observed that the rule with the largest amount of potentiation leads to faster receptive field plasticity. This rule can be associated with the action of noradrenaline on visual cortex, for example. This provides a mechanistic understanding of why noradrenaline can be important to receptive field plasticity in cortical areas, as seen in Martins and Froemke (Martins and Froemke, 2015).

Finally, we asked whether the modulation of presynaptic activity is equivalent to the modulation of plasticity. Our analysis suggests that the upregulation of learning rates can lead to faster learning. However, for low presynaptic activity, strong synaptic weights learn relatively faster than weak weights, regardless of the learning rate amplitude. Since strong weights become stronger, we see a sharpening of receptive field tuning. Upregulating activity, on the other hand, can lead to a scenario in which weak weights learn relatively faster than strong weights. This indicates that modulation of presynaptic activity could lead to a weakening of receptive fields by broadening their tuning.

In this perspective article, we illustrate some of the potential effects that neuromodulators can have in cortical plasticity. By assuming simple neuromodulator-meditated modifications of learning rules, we see interesting differences in the outcomes of receptive field development and adaptation. In all our simulations, we have only used pair-based spike-timing dependent plasticity rules (Gerstner et al., 1993, 1996; Abbott and Blum, 1996; Bi and Poo, 1998; Zhang et al., 1998; Roberts, 1999; Abbott and Nelson, 2000; Mehta et al., 2000; Song et al., 2000; Sjöström et al., 2001; Caporale and Dan, 2008). This allowed us to explore a wide range of different possibilities within a frequently explored and well described framework. Some of the behaviors shown in this perspective article can also be explored analytically, using established techniques of plasticity in feedforward network (Kempter et al., 1999; Song et al., 2000; van Rossum et al., 2000; Kempter et al., 2001; Rubin et al., 2001; Câteau and Fukai, 2003; Izhikevich and Desai, 2003; Zhu et al., 2006; Burkitt et al., 2007; Gilson et al., 2009, 2010; Gjorgjieva et al., 2011; Ocker et al., 2015; Babadi and Abbott, 2016).

Experimental exploration of the effects of neuromodulation in cortical plasticity is a rapidly growing topic of interest. However, the precise effects of neuromodulators in neuronal networks remain unclear, and further experimental data is required. More biologically detailed rules can be explored as descriptions of the effect of neuromodulation on cortical plasticity come to light. Previous voltage-dependent or calcium-dependent models of synaptic plasticity may be modified in this regard (Senn et al., 2001; Shouval et al., 2002; Pfister and Gerstner, 2006; Clopath et al., 2010; Graupner and Brunel, 2012). The effects of neuromodulators under these complex learning rules could be even vaster than for those studied in this perspective article. Moreover, we limited our perspective to a qualitative analysis. With more experimental data and more detailed models, it would be possible to extend this study to a quantitative description.

Synaptic connections in young animals are thought to be highly plastic and are associated with a state of unbalanced excitation and inhibition. In this case, neuromodulation might not be needed for plasticity. However, in adults, experiments indicate that neuromodulation is necessary to open a window of plasticity. We illustrate here that this can be done either by modulating the learning rule (by gating learning) or by modulating neuronal activity (e.g., by disinhibition—Clopath et al., 2016). However, these two scenarios do not yield similar plasticity outcomes in our computational model. In the future, it would be interesting to have more experimental data on how behavior - mediated by neuromodulation - affects both the learning rule and neuronal activity. These insights can then be fed back to computational models to further understand their functional implications.

## 4. Methods

See Supplementary Material. The code is posted in ModelDB.

## Author contributions

VP and CC planned the research, VP performed the simulations and wrote the paper.

## Funding

This work was supported by CAPES Foundation, process n. 99999.001758/2015-02, by EPSRC, the Google Faculty Award, the Leverhulme Trust and the Wellcome Trust.

### Conflict of interest statement

The authors declare that the research was conducted in the absence of any commercial or financial relationships that could be construed as a potential conflict of interest.
