# Supplementary material for: The Role of Neuromodulators in Cortical Plasticity. A Computational Perspective
Source: Front Synaptic Neurosci. 2017 Jan 10;8:38. doi: 10.3389/fnsyn.2016.00038 (PMC5222801; doi:10.3389/fnsyn.2016.00038)
Supplement: Supplementary file 1 [file DataSheet1.pdf]

Supplementary Material: The role of neuromodulators in cortical plasticity. A computational perspective.

Victor Pedrosa and Claudia Clopath*
*Correspondence:
Claudia Clopath
c.clopath@imperial.ac.uk

1 SUPPLEMENTARY METHODS

1.1 Neuron model

For all our simulations we use the leaky integrate-and-fire neuron model (Stein (1967); Gerstner and Kistler (2002)). In this model, the membrane potential of a neuron is described by

$$\tau_m \frac{du}{dt} = -(u - u_{rest}) + RI(t) \,, \tag{1}$$

where u_{rest} denotes the membrane voltage at rest, R denotes the membrane resistance, $I(t)$ denotes the external current and τ_m denotes the membrane time constant. If the membrane potential reaches a threshold u_{th} at time $t^{(f)}$, the membrane potential is reset to u_{reset} and we call $t^{(f)}$ the firing time. After being reset, the membrane potential follows equation 1 again.

The term $I(t)$ takes into account all of the current being injected into a neuron; these can be from an external source (e.g. an electrode) or from other neurons. When a neuron fires, it propagates a current to all other connected neurons. In order to model this current, we assume that the conductance between a presynaptic neuron j and a postsynaptic neuron i increases instantaneously every time the presynaptic neuron fires, and decays exponentially otherwise:

$$g_j \to g_j + 1 \qquad \text{if } j \text{ fires and} \tag{2}$$

$$\frac{dg_j}{dt} = -g_j/\tau_{syn} \qquad \text{otherwise,} \tag{3}$$

where τ_{syn} is the synaptic time constant. The synaptic current is then calculated through

$$I^{syn}(t) = -w_{ij} g_j (u - E^{syn}) \,, \tag{4}$$

where w_{ij} is the synaptic weight from neuron j to neuron i and E^{syn} is the synaptic reversal potential.

1.2 Input signals

Each stimulus is represented by a filtered gaussian noise, with a time constant of $\tau_{filt} = 50$ ms, from which we subtract a constant c, then rectify all negative values to zero and multiply by a constant ν_j (to

control the mean), resulting in a function $s_j(t)$ (a signal, or stimulus). In summary,

$$s_j(t) = \nu_j[f_j(t) - c]_+ \,, \tag{5}$$

$$\tau_{filt}\frac{df_j(t)}{dt} = f_j(t) - \xi_j(t) \,, \tag{6}$$

where $\xi_j(t)$ is a Gaussian white noise. The constant c controls the lifetime sparseness of the signals (Franco et al. (2007)), controlling how well the postsynaptic neuron can differentiate between two presynaptic neurons. When all the stimuli are equally represented, $\nu_j = m$ for all j. When one of the stimuli is over-represented, the corresponding value of ν_j is increased.

We assume that all input neurons received contributions from all stimuli with different intensities. For each neuron, we associated a weighted sum of the stimuli, $p_i(t) = \sum_j T_{ij}s_j(t)$, where T_{ij} is a tuning strength defined from a Gaussian distribution: $T_{ij} \propto \exp\left(-(i-j)^2/2\sigma^2\right)$ and it is normalized such that $\sum_j T_{ij} = 1$. The parameter $\sigma = 1$ is the tuning width. The function $p_i(t)$ is then defined as the firing probability of input neuron i.

See diagram in supplementary figure 1.

1.3 Synaptic plasticity model (pair-based)

According to the STDP rule we use, the synaptic weight between a presynaptic neuron j and a postsynaptic neuron i evolves following (Kistler and van Hemmen (2000); Gerstner and Kistler (2002))

$$\frac{d}{dt}w_{ij}(t) = A_- S_j(t)\int_0^\infty \mathrm{e}^{-s/\tau_-} S_i(t-s)ds + A_+ S_i(t)\int_0^\infty \mathrm{e}^{-s/\tau_+} S_j(t-s)ds \,, \tag{7}$$

where $S_j = \sum_f \delta\left(t - t_j^{(f)}\right)$ and $S_i = \sum_f \delta\left(t - t_i^{(f)}\right)$ are pre- and postsynaptic spike trains, respectively, A_- is the depression amplitude, A_+ is the potentiation amplitude, τ_- is the depression time constant and τ_+ is the potentiation time constant. This rule takes into account only pairs of pre-post or post-pre activity and therefore can be summarized by the effect of only one pair (known as a learning window),

$$\Delta w_{ij}(t) = \begin{cases} A_+ \exp(-t/\tau_+) & \text{if } t < 0 \\ A_- \exp(-t/\tau_-) & \text{if } t > 0 \end{cases} \,, \tag{8}$$

where $t = t_j^{(f)} - t_i^{(f)}$ is the difference between the postsynaptic and the presynaptic firing times. In order to implement this rule, we define a presynaptic trace $\bar{x}_j$ and a postsynaptic trace $\bar{y}_i$ that is incremented by 1 for each pre or postsynaptic spike, respectively, and decay otherwise following

$$\begin{aligned} \bar{x}_j &\to \bar{x}_j + 1 && \text{if presynaptic neuron } j \text{ fires and} \\ \tau_+\frac{d\bar{x}_j}{dt} &= -\bar{x}_j && \text{otherwise,} \end{aligned} \tag{9}$$

and

$$\begin{aligned} \bar{y}_i &\to \bar{y}_i + 1 && \text{if postsynaptic neuron } i \text{ fires and} \\ \tau_-\frac{d\bar{y}_i}{dt} &= -\bar{y}_i && \text{otherwise.} \end{aligned} \tag{10}$$

The synaptic weight w_{ij} is then updated by the following:

$$\begin{aligned} w_{ij}(t) &\rightarrow w_{ij}(t) + A_{-}\bar{y}(t) \qquad \text{if } t = t^{pre}, \\ w_{ij}(t) &\rightarrow w_{ij}(t) + A_{+}\bar{x}(t) \qquad \text{if } t = t^{post}. \end{aligned} \tag{11}$$

Synaptic weights were also bounded between 0 and 2: $0 < w < 2$.

1.4 Synaptic plasticity model (triplet)

In supplementary figure 3, we use the triplet model (Pfister and Gerstner (2006)) to update the synaptic weights. We first consider two detectors of presynaptic events, r_1 and r_2, and two detectors of postsynaptic events, o_1 and o_2. These variables evolve according to the following:

$$\begin{aligned} \frac{dr_1(t)}{dt} &= -\frac{r_1(t)}{\tau_+} \qquad \text{and if } t = t^{pre} \text{ then } r_1 \rightarrow r_1 + 1, \\ \frac{dr_2(t)}{dt} &= -\frac{r_2(t)}{\tau_x} \qquad \text{and if } t = t^{pre} \text{ then } r_2 \rightarrow r_2 + 1, \\ \frac{do_1(t)}{dt} &= -\frac{o_1(t)}{\tau_-} \qquad \text{and if } t = t^{post} \text{ then } o_1 \rightarrow o_1 + 1, \\ \frac{do_2(t)}{dt} &= -\frac{o_2(t)}{\tau_y} \qquad \text{and if } t = t^{post} \text{ then } o_2 \rightarrow o_2 + 1, \end{aligned} \tag{12}$$

where τ_+ and τ_x are time constants for presynaptic events and τ_+ and τ_y are time constants for postsynaptic events. The change in the synaptic weight w_{ij} is then calculated through

$$\begin{aligned} w_{ij}(t) &\rightarrow w_{ij}(t) + o_1(t)\left[A_2^- + A_3^- r_2(t-\epsilon)\right] \qquad \text{if } t = t^{pre}, \\ w_{ij}(t) &\rightarrow w_{ij}(t) + r_1(t)\left[A_2^+ + A_3^+ o_2(t-\epsilon)\right] \qquad \text{if } t = t^{post}, \end{aligned} \tag{13}$$

where A_2^- and A_2^+ denote the amplitude of synaptic weight change for post-pre and pre-post events, respectively. A_3^- and A_3^+ denote the amplitude of depression and potentiation, respectively, for the triplet term.

1.5 Parameters and simulations

1.5.1 Figures 1C - 1E

Our feedforward network consisted of 100 presynaptic neurons and one postsynaptic neuron. All the weights were initialized to 0.2 and the network was run for 1000 s with a time step of 1 ms. All the numerical parameters can be seen in supplementary table 1. In figure 1C, rule 1 was modified by increasing the amplitude for depression by 2%.

1.5.2 Figures 1F - 1K

The feedforward network consisted of 10 presynaptic neurons and one postsynaptic neuron. The weights were initialized by assuming an initial receptive field tuned to input 7. All the curves are calculated from an average over 200 trials. The shading areas represent one standard deviation from the mean. For figures 1F and 1G, the network runs for 20 s. For figures 1I and 1J, the network runs for 80 s. All other numerical parameters can be found in supplementary table 1.

1.5.3 Figures 2A, 2B, 2D and 2E

The feedforward network consisted of one presynaptic neuron and one postsynaptic neuron. All the parameters used in these simulations can be found in supplementary table 2. The network was simulated for 100 s. For each value of the synaptic weight, w, the total change in synaptic weight was calculated as the sum of synaptic weight changes in each time step (1 ms). However, the changes were not applied to the weights to ensure that the calculated synaptic change was specific to each value of w. Therefore, the calculated value of Δw is the effective change in synaptic weight. The curves show an average over 200 trials.

1.5.4 Figure 2C

The feedforward network consisted of 10 presynaptic neurons and one postsynaptic neuron. All the parameters used in these simulations can be found in supplementary table 2. Weights were initialized assuming an initial receptive field tuned to inputs 5 and 6. The final receptive field was calculated for $\nu = 1$ Hz and two values of the learning rate: $\alpha = 0.02$ (large) and $\alpha = 0.01$ (small). The initial and final receptive fields were re-scaled by dividing all the tuning curves by their respective maximum weights. Curves show an average over 50 trials.

1.5.5 Figure 2F

The feedforward network consisted of 10 presynaptic neurons and one postsynaptic neuron. All the parameters used in these simulations can be found in supplementary table 2. Weights were initialized assuming an initial receptive field tuned to inputs 5 and 6. The final receptive field was calculated for $\alpha = 0.02$ Hz and two values of presynaptic activity: $\nu = 10$ Hz (large) and $\nu = 1$ Hz (small). The initial and final receptive fields were re-scaled by dividing all the tuning curves by their respective maximum weights. Curves show an average over 50 trials.

1.5.6 Supplementary figure 2

The feedforward network consisted of 10 presynaptic neurons and one postsynaptic neuron. All the parameters used in these simulations can be found in supplementary table 2. Weights were initialized assuming an initial receptive field tuned to inputs 5 and 6. The final receptive field was calculated for the following values of learning rate (α) and presynaptic activity (ν): $\alpha \downarrow= 0.01$, $\alpha \uparrow= 0.02$, $\nu \downarrow= 1$ Hz, $\nu \uparrow= 10$ Hz. The initial and final receptive fields were re-scaled by dividing all the tuning curves by their respective maximum weights. Curves show an average over 50 trials.

1.5.7 Supplementary figure 3A

The synaptic weights were updated following a triplet rule. The feedforward network consisted of 10 presynaptic neurons and one postsynaptic neuron. All the parameters used in these simulations can be found in supplementary table 2. Weights were initialized assuming an initial receptive field tuned to inputs 5 and 6. The final receptive field was calculated for $\nu = 1$ Hz and two values of the learning rate: $\alpha = 0.02$ (large) and $\alpha = 0.01$ (small). The initial and final receptive fields were re-scaled by dividing all the tuning curves by their respective maximum weights. Curves show an average over 50 trials.

1.5.8 Supplementary figure 3B

The synaptic weights were updated following a triplet rule. The feedforward network consisted of 10 presynaptic neurons and one postsynaptic neuron. All the parameters used in these simulations can be found in supplementary table 2. Weights were initialized assuming an initial receptive field tuned to inputs

5 and 6. The final receptive field was calculated for $\alpha = 0.02$ Hz and two values of presynaptic activity: $\nu = 5$ Hz (large) and $\nu = 1$ Hz (small). The initial and final receptive fields were re-scaled by dividing all the tuning curves by their respective maximum weights. Curves show an average over 50 trials.

2 SUPPLEMENTARY TABLES AND FIGURES

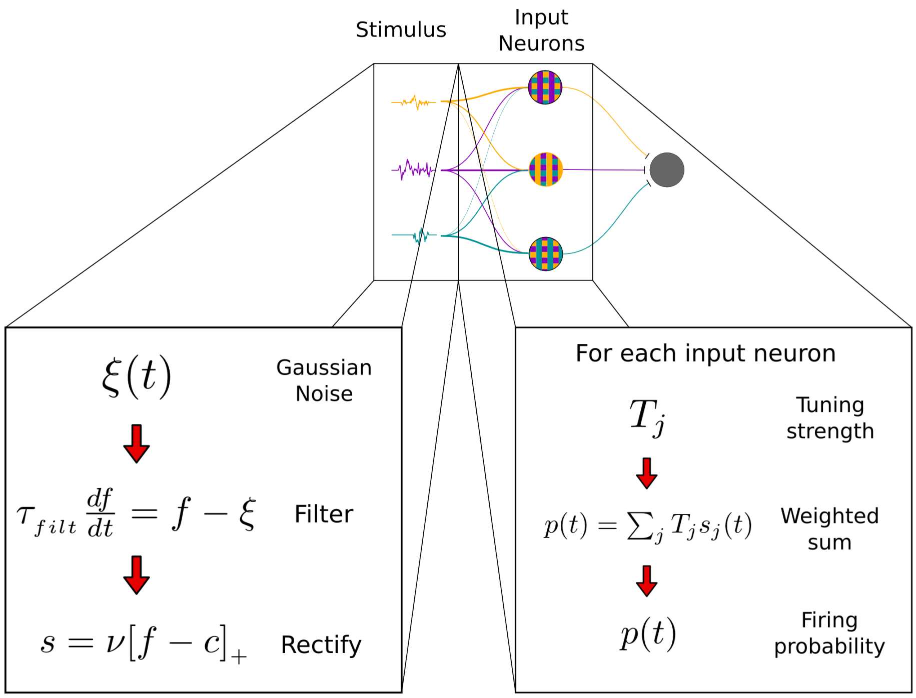

Supplementary Figure 1. Generation of presynaptic spike trains. The procedure used to generate the firing probability of each input neuron can be split into two steps: (i) generation of N independent rectified time-filtered Gaussian white noise (left box); (ii) weighted sum of the N signals generated in the first step (right box). The set of tuning strengths for each neuron is defined such that input neuron i is tuned to signal i.

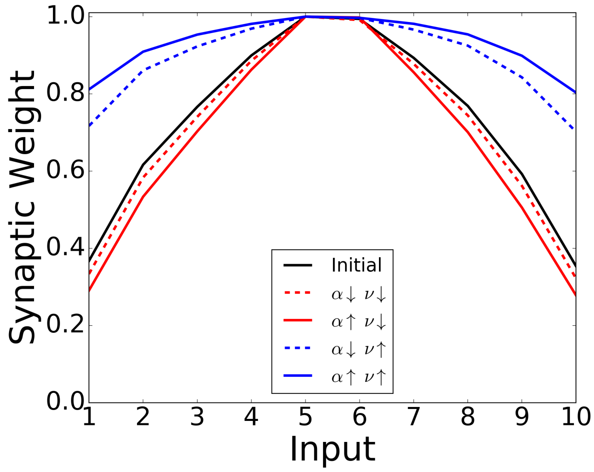

Supplementary Figure 2. Receptive field plasticity under neuronal activity and/or learning rate modulation. Synaptic weights for a feedforward network with 10 presynaptic neurons and one postsynaptic neuron. The final synaptic weights were calculated for the following values of learning rate (α) and presynaptic activity (ν): $\alpha\downarrow = 0.01$ (dashed lines), $\alpha\uparrow = 0.02$ (solid lines), $\nu\downarrow = 1$ Hz (red curves), $\nu\uparrow = 10$ Hz (blue curves). The initial and final receptive fields were rescaled by dividing all the tuning curves by their respective maximum weights. The effects of both types of modulations are independent.

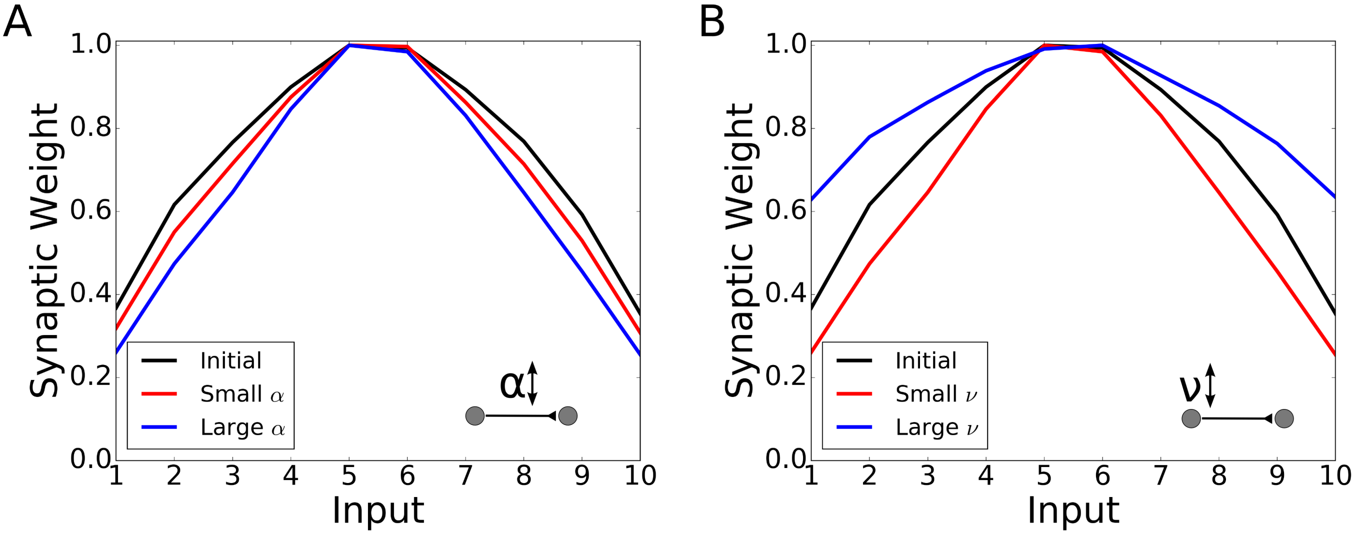

Supplementary Figure 3. Modulation of activity vs modulation of learning rate, using the triplet model (Pfister and Gerstner (2006)). Synaptic weights for a feedforward network with 10 presynaptic neurons and one postsynaptic neuron. The final synaptic weights were simulated for the following values of learning rate (α) and presynaptic activity (ν): **A** $\nu = 1$ Hz, small $\alpha = 0.01$ (red curve), large $\alpha = 0.02$ (blue curve); **B** $\alpha = 0.01$, small $\nu = 1$ Hz (red curve), large $\nu = 5$ Hz (blue curve). In all the figures, the initial and final receptive fields were rescaled by dividing all the tuning curves by their respective maximum weights. The results observed with the triplet model are qualitatively identical to the results observed with the standard pair-based STDP model.

Supplementary Table 1. Parameter summary for simulations in figure 1.

**Neuron Model**		
**Name**	**Value**	**Description**
τ_m	50 ms	Membrane time constant
u_{th}	10 mV	Spiking threshold
u_{rest}	0 mV	Resting potential
E^{syn}	30 mV	Synaptic reversal potential
u_{reset}	0 mV	Value at which the potential is reset after a spike

**Network and Synapse Model**		
**Name**	**Value**	**Description**
N_E (C-E)	100	Size of presynaptic population for figure 1C-E
N_E (F-K)	10	Size of presynaptic population for figure 1F-K
τ_E	15 ms	Decay constant of excitatory conductance
$\bar{g}_E$	1 nS	Peak synaptic conductance

**Plasticity Model**		
**Name**	**Value**	**Description**
τ_{STDP}	8 ms	Decay constant of pre- and post synaptic traces
A_+	$\gamma_+ 3 \times 10^{-4}$	Amplitude of learning rate for pre-post events
A_-	$\gamma_- 3 \times 10^{-4}$	Amplitude of learning rate for post-pre events
γ_+	1, 1, 1, 0	For rules 1, 2, 3 and 4, respectively
γ_-	-1, 1, 0, -1	For rules 1, 2, 3 and 4, respectively

Supplementary Table 2. Parameter summary for simulations in figure 2.

**Neuron Model**		
**Name**	**Value**	**Description**
τ_m	10 ms	Membrane time constant
u_{th}	10 mV	Spiking threshold
u_{rest}	0 mV	Resting potential
E^{syn}	30 mV	Synaptic reversal potential
u_{reset}	0 mV	Value at which the potential is reset after a spike

**Network and Synapse Model**		
**Name**	**Value**	**Description**
N_E	1	Size of presynaptic population for figure 2
N_E	10	Size of presynaptic population for figure 2
τ_E	10 ms	Decay constant of excitatory conductance
$\bar{g}_E$	0.1 nS	Peak synaptic conductance
ν	ν	Presynaptic firing rate ($\nu_0 = 10$ Hz)

**Plasticity Model (pair-based)**		
**Name**	**Value**	**Description**
τ_{STDP}	8 ms	Decay constant of pre- and post synaptic traces
A_+	$+\alpha$	Amplitude of learning rate for pre-post events ($\alpha_0 = 5 \times 10^{-4}$)
A_-	$-\alpha$	Amplitude of learning rate for post-pre events ($\alpha_0 = 5 \times 10^{-4}$)

**Plasticity Model (triplet)**		
**Name**	**Value**	**Description**
$\tau_{+/-}$	8 ms	Decay constant of pre- and post synaptic traces (r_1 and o_1)
$\tau_{x/y}$	10 ms	Decay constant of pre- and post synaptic traces (r_2 and o_2)
A_2^+	$+\alpha$	Amplitude of learning rate for pre-post events
A_2^-	$-\alpha$	Amplitude of learning rate for post-pre events
A_3^+	$+\alpha$	Amplitude of the triplet term for potentiation
A_3^-	$-\alpha$	Amplitude of the triplet term for depression

REFERENCES

Franco, L., Rolls, E. T., Aggelopoulos, N. C., and Jerez, J. M. (2007). Neuronal selectivity, population sparseness, and ergodicity in the inferior temporal visual cortex. *Biological Cybernetics* 96, 547–560. doi:10.1007/s00422-007-0149-1

Gerstner, W. and Kistler, W. (2002). *Spiking Neuron models* (Cambridge University Press)

Kistler, W. M. and van Hemmen, J. L. (2000). Modeling synaptic plasticity in conjuction with the timing of pre- and postsynaptic action potentials. *Neural computation* 12, 385–405. doi:10.1162/089976600300015844

Pfister, J.-P. and Gerstner, W. (2006). Triplets of spikes in a model of spike timing-dependent plasticity. *The Journal of neuroscience : the official journal of the Society for Neuroscience* 26, 9673–82. doi:10.1523/JNEUROSCI.1425-06.2006

Stein, R. B. (1967). Some Models of Neuronal Variability. *Biophysical Journal* 7, 37–68. doi:10.1016/S0006-3495(67)86574-3
